# L-type calcium channels and neuropsychiatric diseases: Insights into genetic risk variant-associated genomic regulation and impact on brain development

**DOI:** 10.1080/19336950.2023.2176984

**Published:** 2023-02-19

**Authors:** Madelyn R. Baker, Andrew S. Lee, Anjali M. Rajadhyaksha

**Affiliations:** a Neuroscience Program, Weill Cornell Graduate School of Medical Sciences, New York, USA; b Department of Pharmacology, Weill Cornell Medicine, New York, USA; c Developmental Biology Program, Sloan Kettering Institute, New York, USA; d Pediatric Neurology, Department of Pediatrics, Weill Cornell Medicine, New York, USA; e Feil Family Brain and Mind Research Institute, Weill Cornell Medicine, New York, USA; f Weill Cornell Autism Research Program, Weill Cornell Medicine, New York, USA

**Keywords:** CACNA1C, CACNA1D, Cav1.2, Cav1.3, neuropsychiatric

## Abstract

Recent human genetic studies have linked a variety of genetic variants in the *CACNA1C* and *CACNA1D* genes to neuropsychiatric and neurodevelopmental disorders. This is not surprising given the work from multiple laboratories using cell and animal models that have established that Ca_v_1.2 and Ca_v_1.3 L-type calcium channels (LTCCs), encoded by *CACNA1C* and *CACNA1D*, respectively, play a key role in various neuronal processes that are essential for normal brain development, connectivity, and experience-dependent plasticity. Of the multiple genetic aberrations reported, genome-wide association studies (GWASs) have identified multiple single nucleotide polymorphisms (SNPs) in *CACNA1C* and *CACNA1D* that are present within introns, in accordance with the growing body of literature establishing that large numbers of SNPs associated with complex diseases, including neuropsychiatric disorders, are present within non-coding regions. How these intronic SNPs affect gene expression has remained a question. Here, we review recent studies that are beginning to shed light on how neuropsychiatric-linked non-coding genetic variants can impact gene expression via regulation at the genomic and chromatin levels. We additionally review recent studies that are uncovering how altered calcium signaling through LTCCs impact some of the neuronal developmental processes, such as neurogenesis, neuron migration, and neuron differentiation. Together, the described changes in genomic regulation and disruptions in neurodevelopment provide possible mechanisms by which genetic variants of LTCC genes contribute to neuropsychiatric and neurodevelopmental disorders.

## Overview of *CACNA1C* AND *CACNA1D* genetic risk variants

L-type calcium channels (LTCCs) are a subgroup of voltage-gated calcium channels that include four members: Ca_v_1.1–1.4. The most prominent forms in the brain are Ca_v_1.2 and Ca_v_1.3, encoded by the genes *CACNA1C* and *CACNA1D*, respectively [1]. Recent genetic studies have implicated *CACNA1C* and *CACNA1D* in neurodevelopmental and neuropsychiatric diseases (with neurodevelopmental origins [2–5]). The first link of LTCCs to human brain disease was the discovery of the Ca_v_1.2 single-point mutation G406R in Timothy Syndrome, a multi-organ disorder that includes cardiac and neurological autism spectrum disorder (ASD)-like behavioral symptoms [6]. Soon after, G402S was identified [7], followed by I1166T [8], both manifesting ASD-like symptoms. More recently, multiple additional Ca_v_1.2 mutations have been identified in patients with a variety of symptoms, including new mutations linked to both cardiac and ASD symptoms, while others not causing ASD [9]. Studies have begun to examine the impact of the various mutations on Ca_v_1.2 channel properties to gain deeper knowledge on possible mechanisms that result in the cardiac versus brain symptoms [10]. We direct readers to excellent reviews by Marcantoni et al. 2020 [9] and Herold et al. 2023 [11] on this topic. In the neurodevelopmental section below, we focus on Ca_v_1.2 mutations that have been linked to ASD phenotypes in patients. Similar to Ca_v_1.2, missense coding genetic variants in *CACNA1D* (Ca_v_1.3) have been linked to ASD and intellectual disability [12]. These disease-linked mutations have direct effects on channel gating and are believed to underlie the observed ASD-associated phenotypes. We direct readers to the excellent reviews by Ortner et al. 2020 [12] and Ortner 2023 [13] on Ca_v_1.3 mutations and their impact on channel properties.

In addition to the coding variants, non-coding single nucleotide polymorphisms (SNPs) (Box 1) in *CACNA1C* have been found in Genome-Wide Association Studies (GWASs) (Box 1) to be associated with bipolar disorder (BD) [14–17], schizophrenia (SCZ) [18], major depressive disorder (MDD) [19], ASD [20], and attention deficit hyperactivity disorder (ADHD) [21]. Similarly, SNPs in *CACNA1D* have been linked to BD, SCZ, ASD, and intellectual disability (ID) [22–24]. The majority of these SNPs are present in intronic regions, particularly for *CACNA1C*. This is in accordance with the growing body of literature establishing that SNPs associated with complex diseases, such as neuropsychiatric disorders are present within non-coding genomic regions [25]. The mechanisms by which these SNPs impact gene expression, and in turn channel function, have remained a question. Since many neuropsychiatric disease-associated SNPs are located in these non-coding regions of the genome, research focus has shifted to examining the influence of intronic SNPs in the context of broader genomic regulation [26–28]. This includes studies on how disease-linked SNPs present within cis-regulatory elements that include enhancers and promoters (Box 1), impact chromatin at a three-dimensional (3D) level and subsequently impact gene expression (as discussed in the next section).

**Box 1.** Definitions of terms

Term	Definition
**Epigenetics section**	
Single nucleotide polymorphism (SNP)	A substitution at a single nucleotide of DNA (a genetic variant) that can be used to examine its association to a disorder
Genome wide associate study (GWAS)	A study connecting SNPs across the genome in a large population to complex diseases
Chromosome conformation capture	A technique to study the 3D spatial conformation of chromatin by finding interactions between genomic regions
Linkage disequilibrium	The association of specific alleles in two or more genomic loci, occurring nonrandomly
Promoters	The region of DNA that binds factors necessary for the initiation of transcription of a gene
Alternative promoter	An alternate transcriptional start site that influences transcriptional regulation through starting transcription at different points to include different exons
CpG Island	A region of DNA that contains a high frequency of CpG sites; they have stable methylation, generally in the unmethylated state.
Quantitative trait locus	Loci of DNA that correlate with a trait of a phenotype across a population
Epigenome-Wide Association Studies (EWAS)	A study connecting an epigenetic variant (often DNA methylation) with a phenotype
Long-range regulatory elements	A genomic element that impacts gene expression through long-range interactions with the promoter
Enhancer (and active vs poised)	A region of DNA that acts as a regulatory element through increasing transcription of a gene; active enhancers are actively involved in regulating transcription while poised enhancers modulate expression only in response to other cues
Repressor	A DNA-binding protein that decreases the expression of a gene through blocking the binding of RNA polymerase or other factors for transcription
Silencer	A region of DNA that repressors bind to that act as a regulatory element through decreasing gene expression
Insulator	A region of DNA that prevents the interaction between regulatory regions of DNA
Cis-regulatory elements	Regions of non-coding DNA that regulate transcription of nearby genes
DNA methylation	The addition of a methyl group to the cytosine of a CG dinucleotide (termed CpG site) to form 5-methylcytosine, generally, a repressive mark
Histone modifications	An addition of a chemical group (i.e. acetylation, methylation, phosphorylation, ubiquitination) to a histone that can impact gene expression through impacting chromatin structure
**Neurodevelopment section**	
*In utero* electroporation	A method to introduce a plasmid DNA into developing mouse brains, while the embryos are still alive in the uterus.
Human induced pluripotent stem cell (iPSC)	Cells derived from human skin or blood cells by reprogramming them back into a pluripotent state that allows the generation of any type of human cell.
Cerebral organoids	An artificially grown (from iPSCs) miniature organ that resembles the brain *in vitro*. See Pasca et al., 2022 [29] for more details.
Forebrain assembloids	Brain organoids that are a combination of multiple brain organoids that represent different brain regions/cell types in 3D culture. See Pasca et al., 2022 [29] for more details.
Tet-OFF TRE/rtTA system	A system to silence gene expression (OFF) by treatment with tetracycline or tetracycline-derivatives (e.g. doxycycline). Tetracycline or tetracycline-derivatives binds to rtTA (which is controlled by a specific promoter) which inhibits the binding of rtTA to Tet/TRE, silencing gene expression.

In this review, we will discuss new literature on how neuropsychiatric-linked non-coding genetic variants can impact gene expression via regulation at the genomic level, with a focus on *CACNA1C*, which has been studied more over the last several years compared to *CACNA1D*. We additionally review recent findings on how aberrant Ca_v_1.2 and Ca_v_1.3 channel expression or function can impact brain development as studied in both *in vitro* and *in vivo* models of brain development. Readers are directed to previous reviews that have covered *CACNA1C* and *CACNA1D* genetic variants, animal studies, molecular signaling mechanisms of these channels, and their contribution to neuropsychiatric-related cognitive domains [9,22,23,30–34].

## Intronic non-coding SNPs and genomic regulation

**Figure 1.**
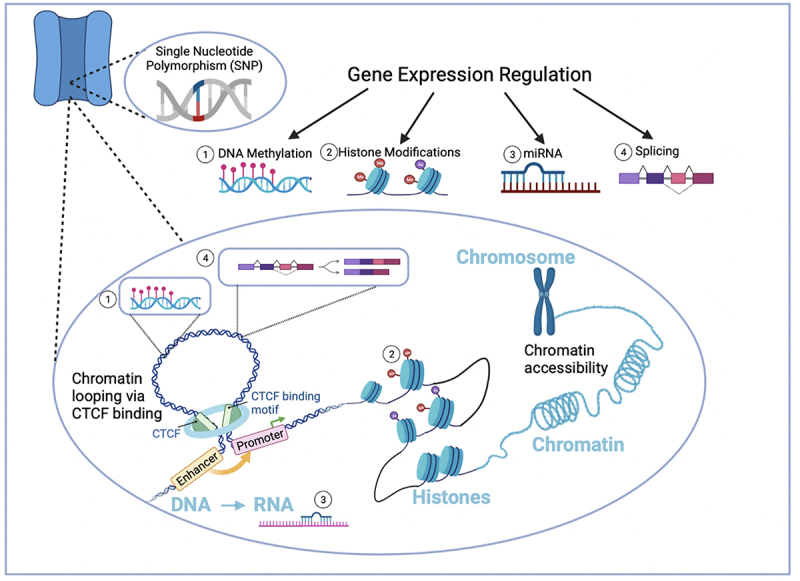
Gene expression regulatory processes at the level of chromatin that can be disrupted by disease-associated SNPs as observed in LTCC genes *CACNA1C* and *CACNA1D*. Gene expression can be regulated at the level of (1) DNA methylation, (2) histone modifications, (3) miRNA, and (4) splicing. Additionally, structural changes at the genomic level, such as chromatin looping which brings regulatory DNA elements such as enhancers and promoters in close proximity, are involved in activation transcription. The protein CTCF (CCCTC DNA-binding factor) plays a key role in changes in chromatin architecture.

The development of new technologies, such as chromosome conformation capture [35,36] (Box 1), which measures physical interactions in the genome to probe the 3D mammalian genome, has greatly advanced our understanding of the structural and functional dynamics of the genome and how genes are transcribed [37,38]. In eukaryotic cells, DNA is in a compact form, with 146–147 base pairs of DNA wrapped around an octamer of histones, making up nucleosomes that are further condensed into chromatin, which is what makes up a chromosome [39–41] (Figure 1). It is now clear that although genetic information is encoded in DNA’s linear sequences, transcriptional regulation is dictated by the 3D hierarchical organization of chromatin bringing together different DNA domains across multiple kilobases in close proximity to each other, to activate gene expression (Figure 1; see Fujita et al., 2022 [42] for review).

With the majority of the genome consisting of non-coding DNA and the complexity of 3D chromatin structure, studies have shifted to identifying transcriptional regulatory regions at the gene level. This has allowed looking at how 3D interactions are disrupted by non-coding genetic variants in complex diseases, including neuropsychiatric diseases [43]. Recent large-scale initiatives such as the ENCODE (ENCyclopedia of DNA Elements) consortium have found that non-coding intronic regions are rich in regulatory elements including enhancers, repressors, silencers, and insulators (Box 1) that can influence gene expression. These intronic elements interact with cis-regulatory elements, such as promoters (Box 1), and transcription factor binding sites classically present at the 5’ end of genes, through long-range structural chromatin modifications (Figure 1) [38,44]. Many disease-linked SNPs are present within these non-coding regions of the DNA that have the potential to disrupt normal transcriptional mechanisms [45].

Other mechanisms that occur either before or in tandem with the structural chromatin changes include epigenetic changes such as DNA methylation and histone modifications (Box 1). At the level of the RNA transcript, microRNAs and alternative splicing further impact mRNA levels or types of transcripts generated. Non-coding SNPs can dysregulate any of the mechanisms described above, in turn impacting gene expression. Below we review some new literature characterizing the genomic landscape of the *CACNA1C* gene and the interaction of non-coding SNPs with genomic elements to influence gene expression. Such knowledge is proving to be critical for understanding the genetics of complex disorders, such as genetic findings from GWASs of neuropsychiatric disorders [46–48].

### Chromatin looping

One of the structural changes that promotes transcriptional activation is the looping of DNA, referred to as chromatin looping, which regulates transcription by changing the proximity of different regulatory elements [49]. For example, a chromatin loop can bring an enhancer that is thousands of base pairs away from the promoter of the gene in close proximity to increase gene expression (Figure 1). Thus, intronic SNPs within these regulatory regions can disrupt chromatin looping by affecting the accessibility of DNA-binding factors necessary for chromatin looping [50–52]. Disruptions in chromatin looping have been implicated in neurological disorders, such as schizophrenia, Alzheimer’s disease, and addiction, as reviewed by Behrends and Engmann [51].

Specific to mouse *Cacna1c*, a few studies have highlighted the importance of chromatin looping in transcriptional activation and the impact of intronic SNPs. A demonstration for the importance of looping has come from the study of the zinc finger protein CCCTC DNA-binding factor (CTCF), a well-defined regulator of chromatin architecture. Mice with a knockout of CTCF in cardiomyocytes have a loss of almost all global chromatin loops, which in turn leads to decreased *Cacna1c* expression in the heart [53]. Though only demonstrated in the heart, this regulation of *Cacna1c* expression through chromatin loops could occur similarly in the brain.

Important to the genomic regulation of human *CACNA1C*, the third intron of *CACNA1C* contains SNPs linked to multiple neuropsychiatric diseases [21]. One of the first observations that disease-associated SNPs could impact gene regulation came from the work of Roussos et al. (2014) showing that multiple SCZ-associated SNPs in the third intron are in an enhancer region that interacts with the proximal gene promoter [54]. Using chromosome conformation capture (Box 1; 3C-seq and 4C-seq;), which characterizes the spatial organization of chromatin and finds interactions between genomic loci [55], they identified that a region in intron 3 interacted with the *CACNA1C* promoter through chromatin looping [54]. The intronic region contained the strongest SCZ risk SNP (rs1006737) and majority of the *CACNA1C* SNPs that are in high linkage disequilibrium (Box 1). These SNPs disrupted chromatin looping, decreasing transcriptional activity and reducing *CACNA1C* mRNA levels. This demonstrated a mechanism by which intronic SNPs could disrupt chromatin looping and gene expression [54]. They observed this in human post-mortem SCZ brains, human induced pluripotent cells, HEK, and SKN-SH cell lines [54]. Additional regions have been found in the third intron of *CACNA1C* with enhancer activity, which could also regulate transcriptional activity of the gene [56]. Eckart et al. (2016) have similarly demonstrated with chromatin conformation capture that there is an interaction of a SCZ-associated region in intron 3, with the *CACNA1C* promoter and other potential regulatory regions that influence gene expression [57]. This region within intron 3 contains 16 SNPs that are in linkage disequilibrium with the previously mentioned SCZ risk SNP rs1006737 that could also disrupt chromatin looping. These studies demonstrate that disruption of chromatin looping required for normal gene expression could be one way in which intronic SNPs lead to changes in *CACNA1C* gene expression that could contribute to neuropsychiatric disease.

### Epigenetics

Epigenetic modifications regulate the genome and have many downstream effects through altering gene expression without changing the DNA sequence [58,59]. These modifications include classical chromatin altering mechanisms, such as DNA methylation and post-translational modifications of histones, as well as non-classical mechanisms, such as regulation by non-coding microRNAs (Box 1). All of these modes of genome regulation have been associated with neuropsychiatric disorders, including MDD, ASD, Fragile X syndrome, Rett syndrome, BD, and SCZ [60].

### A. DNA methylation

DNA methylation is the addition of a methyl group to the cytosine of a CG dinucleotide (termed CpG site) to form 5-methylcytosine [61], and is one of the most-studied epigenetic mechanisms. Alterations of DNA methylation levels can play a direct role in regulating gene expression [62,63], alternative splicing [64–66], and alternative promoter (Box 1) usage [67,68]. DNA methylation is a dynamic process within cells that is regulated by DNA methyltransferases that are cytosine methylases belonging to a conserved family of proteins [69]. DNMT1 is the maintenance methyltransferase, responsible for copying DNA methylation from the old DNA strand to the new one during cell division, which in the brain occurs during development prior to neural stem cells becoming mature neurons, while DNMT3A and DNMT3B are responsible for *de novo* activity-dependent DNA methylation [70–72]. A study in mouse embryonic cells overexpressing DNMT1 showed lower levels of *Cacna1c* mRNA levels [73], demonstrating that altered level of DNA methylation can influence transcription of the *Cacna1c* gene.

SNPs can occur either within or nearby CpG sites, disrupting normal DNA methylation, either directly or indirectly via altering genome-level interactions required for transcription [74], as described in previous sections. Differential DNA methylation of the *CACNA1C* gene and the interaction between methylation and SNPs has been observed in BD [75], a disorder with well-characterized *CACNA1C* SNPs [14–17]. When analyzing 169 CpG sites spread across five CpG islands (CGIs; Box 1) in the *CACNA1C* gene using DNA isolated from the blood of BD patients, Starnawska et al. (2016) found that one of the five CGIs studied demonstrated intermediate levels of methylation compared to that of healthy controls [75]. Within this one CGI, five of the six CpG sites showed hypermethylation in patient samples. The study additionally examined the interaction of BD-associated SNPs with the observed hypermethylation, and found that SNP rs2238056 in intron 3 had the strongest methylation quantitative trait locus (Box 1), pointing to a mechanism of how SNPs can interact with DNA methylation status to influence *CACNA1C* gene expression [75].

Differential methylation of genes including *CACNA1C* have also been found in patients with psychosis in an Epigenome-Wide Association Study (EWAS; Box 1) [76]. The CpG at the intronic position cg01833890 in *CACNA1C* was found to be differentially methylated in patients exhibiting psychosis [76]. Similarly, the rs1990322 locus in *CACNA1C* at cg24393317 was significantly associated with PTSD [77] and variable methylation was found between depression-discordant monozygotic twins at position cg10031793 in *CACNA1C* [78]. A methylation quantitative trait locus (significant SNP-CpG methylation association), rs2283291, was also found in *CACNA1C* using DNA isolated from postmortem prefrontal cortex tissue from patients with SCZ [79] and was associated with SCZ in a study of Chinese men [80]. All of these studies, except for the ones looking in postmortem tissue, looked at methylation in DNA isolated from patient blood. Even though it is difficult to know if methylation status in blood cells parallels brain methylation status, these studies suggest that SNPs can influence methylation status of the *CACNA1C* gene, which could alter gene expression levels.

### B. Histone modifications

Post-translational modifications of histones (Figure 1) can affect how the DNA is able to wrap around the histone, changing accessibility to DNA and thus influencing gene transcription [81]. Histone modifications promote altered levels of chromatin condensation, resulting in either “open” (referred to as euchromatin) or “closed” (referred to as heterochromatin) states of chromatin, that increase or decrease transcription, respectively [82,83].

Histone acetylation and methylation are the most studied post-translational modifications. H3 mono-methylation of lysine 4 (H3K4me1) and H3 acetylation of lysine 27 (H3K27ac) are associated with the “open” chromatin state and promote transcription [84,85]. Supporting regulation of *CACNA1C* by histone modifications is the presence of H3K4me1 in an enhancer region within intron 3 of *CACNA1C* [86]. In BD patients, seven disease-associated SNPs are present within this intron 3 enhancer [44] that could modify histone binding and alter *CACNA1C* mRNA expression, as has been reported for schizophrenia-associated SNPs also present within this region [54].

The importance of this *CACNA1C* intron 3 enhancer and histone modification is underscored by the observation that one of the SCZ-associated risk SNP in this region interacts via long-range chromatin looping with the *CACNA1C* proximal promoter [54,57]. The functional significance of this interaction comes from the finding that the promoter region harbors a binding site for the epigenetic regulator, EZH2 (Enhancer of zeste homolog 2) [87]. EZH2 is a histone-lysine N-methyltransferase enzyme that participates in histone methylation by addition of methyl groups to histone H3 at lysine 27, resulting in transcriptional repression. Consistent with EZH2ʹs role as a repressor, *in vitro* studies in the SH-SY5Y cell line have found that EZH2 represses reporter expression of a construct containing the *CACNA1C* promoter. The importance of EZH2 and thus histone modifications to *CACNA1C* in SCZ and BD is further highlighted by the observation that expression analysis from the anterior cingulate cortex region of SCZ and BD patient brains showed highly upregulated EZH2 that correlated with downregulated expression of *CACNA1C* [88]. Thus, EZH2, a histone modifying enzyme, through its actions at the *CACNA1C* promotor, and/or through disrupting interactions with enhancer-containing SNPs, could impact *CACNA1C* expression.

### C. microRNA

MicroRNAs (miRNAs) are a type of short non-coding RNAs, 21–26 nucleotides long, that can regulate gene expression [89]. They do so through binding to complementary mRNA, often at the 3’ untranslated region (UTR) and decrease gene expression by repressing translation or degrading mRNA [89,90]. Converging animal and human studies have identified a key role of microRNAs in neuropsychiatric disorders [91,92].

*CACNA1C* is a known target of a miRNA, microRNA137 (*MIR137*) [93,94], that has been linked to SCZ [95]. In HEK-293T cells, transfection of miR-137 results in decreased *CACNA1C* expression [94,96]. It turns out that *EZH2*, described above, is also a validated target of *MIR137* [97,98], thus it is possible that the miR-137 could also regulate *CACNA1C* indirectly via changes in *EZH2* expression. Functional studies have demonstrated that increasing expression of miR-137 in adult neural stem cells isolated from mice *in vitro* increases cell proliferation, whereas decreasing miR-137 reduces proliferation. Similarly, increasing miR-137 expression in the adult mouse hippocampus *in vivo* led to increased adult hippocampal neurogenesis [99], a mechanism regulated by *cacna1c* [100] and altered in neuropsychiatric disorders [101–103]. In addition, in mouse hippocampal neurons, miR-137 regulates synapse formation, maturation, and transmission [104]. Therefore, aberrations in MIR137 in neuropsychiatric conditions could be due to altered expression of *CACNA1C* and related genes that impacts brain cellular and synaptic mechanisms in the hippocampus. Additionally, another miRNA, miR-4300, which also targets *CACNA1C*, was found to be enriched in copy number variable areas, which is a section of the genome that has a variable number of copies between individuals, in a treatment-resistant SCZ patient [105].

### D. Splicing

Recent studies have underscored alternative splicing as a key molecular process that vastly expands the proteome [106]. Interestingly, dysregulation of alternative splicing has been linked to neuropsychiatric [107–109] and neurodevelopmental disorders [110,111]. Not surprisingly, alternative splicing is regulated by multiple epigenetic influences, including DNA methylation [64,65,112] and disease-linked SNPs [113,114], showing that genetic variants could impact normal splicing and contribute to neuropsychiatric diseases.

Recent developments in long-read sequencing have allowed for the characterization of the deep complexity of human *CACNA1C* splicing, identifying 38 novel exons and 241 novel transcripts [115]. There are variations in splice isoforms across different brain regions, which could help differentiate functional differences in the variants [115]. Because many SNPs are in non-coding regions of *CACNA1C* [25], it reasons that SNPs occurring at splice sites within mRNAs could impact splicing and thus could affect transcriptional and translational regulation of the gene.

One of the clearest examples of the effect of splicing occurs in TS in the *CACNA1C* gene, where the G406R mutation in the alternatively spliced exon 8A of *Cacna1c* leads to aberrant splicing of exons 8 and 8A, which is regulated during cortical development [116]. The G406R mutation prevents a normal developmental switch of exon utilization from exon 8A to exon 8, which leads to Timothy Syndrome 1 and Timothy Syndrome 2, respectively. Below we describe the impact of TS mutations on brain development.

Even though much less studied than *CACNA1C*, changes in the splicing of *Cacna1d* have been reported in a zebrafish model of SCZ [117]. In larval zebrafish, heterozygotes with the splice mutation sa17298 in *Cacna1d* results in 50% reduction of splice variants 201 and 202 [117]. This reduction in splice variants led to “psychosis-like” behaviors, including startle responses to dark flashes and locomotor activity under constant light that could be reversed with antipsychotics, such as risperidone and haloperidol [117]. This finding demonstrated a role for *Cacna1d* in SCZ-relevant behaviors and a potential schizophrenia-like model in zebrafish for studying the role of *Cacna1d* splicing.

## L-type calcium channels and brain development

### Major steps in cortical development

**Figure 2.**
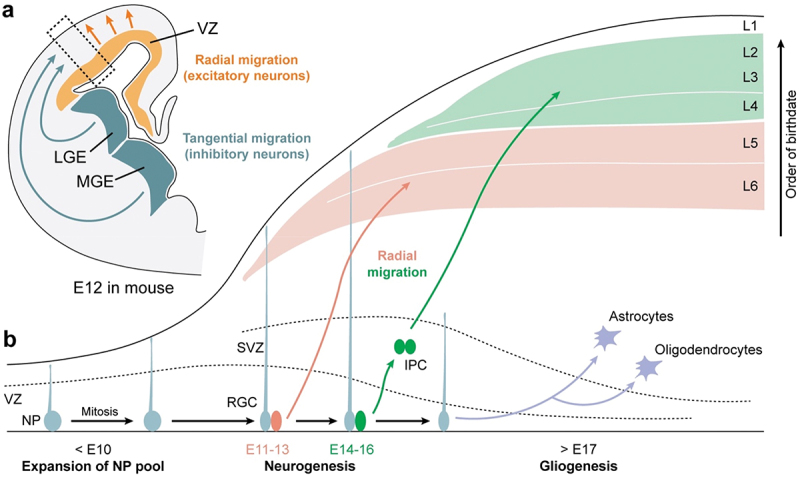
Major steps in mammalian cortical development. (a) Coronal diagram of the embryonic mouse brain at embryonic day 12 (E12). (b) Zoom in of dotted box in (a), showing major steps of neurogenesis and gliogenesis. LGE=lateral ganglionic eminence; MGE=medial ganglionic eminence; NP=neural progenitors; RGC=radial glial cells; SVZ=subventricular zone; VZ=ventricular zone; IPC=intermediate progenitor cells; L=layer.

It is critical that proper genetic programs are recruited for normal brain development. The mammalian central nervous system is comprised of highly interconnected brain regions that carry out a variety of higher cognitive, emotional, and sensorimotor functions that are disrupted in neuropsychiatric and neurodevelopmental disorders [118]. In particular, the cerebral cortex, a widespread sheet of neural tissue at the outermost region of the cerebrum, plays a key role in the execution of complex behaviors [118]. The cerebral cortex consists of six layers, which contain groups of neurons that are genetically, morphologically, neurochemically, and electrophysiologically distinct [118]. Furthermore, each layer makes connections to and from distinct cortical and subcortical regions that allow communication between different brain regions. Given the complexity of the organization and function of the cerebral cortex, it is critical that the six-layer structure, as well as its connections, are properly assembled during development. Establishing the cytoarchitecture and neural circuits of the cerebral cortex, as well as other brain regions, requires numerous developmental processes that need to be temporally and spatially regulated (Figure 2B). The process can be grossly divided into four steps: (1) generation of neurons and glia (neurogenesis and gliogenesis), (2) specification of identity and spatial location of neurons and glia (differentiation and migration), (3) forming connections with other neurons (synaptogenesis, axonogenesis, myelination), (4) experience-dependent maturation of neural circuits.

Genetic mutations that occur in genes that are critical for cortical development can lead to aberrant development of the cerebral cortex layering, circuit wiring, and ultimately, function. These aberrations lead to behavioral impairments and increased susceptibility to neuropsychiatric disorders including SCZ and ASD [15,119–121]. Given the link between LTCCs and neuropsychiatric disorders [2,22,23,34] and the impact of *CACNA1C* and *CACNA1D* mutations on channel function (as in Timothy syndrome [6,8,12] and ASD [24], respectively) and of CACAN1C SNPs on gene expression, it is critical to understand the contribution of LTCCs in cerebral cortex development. Here, we review how perturbations in LTCC expression and/or function impacts different developmental processes.

### Neurogenesis and gliogenesis

The initial step of cortical development is generating the proper number of neurons and glia (astrocytes, oligodendrocytes, and microglia). Based on their function, neurons in the cerebral cortex can be divided into excitatory or inhibitory neurons. Excitatory neurons in the cerebral cortex originate from a layer of neural stem cells above the lateral ventricles known as the ventricular zone (VZ) (Figure 2a), while inhibitory neurons originate from a group of stem cells below the lateral ventricles known as the ganglionic eminence (GE) (Figure 2a). Before embryonic day (E) 10, neural stem cells in the VZ undergo mitosis (i.e. proliferate) to generate a sufficient number of neural progenitors. Thereafter, neural progenitors take an elongated morphology (radial glial cells; RGC, Figure 2b) to form a radial scaffold that guides the newborn neurons to their proper location. RGCs not only help the migration of neurons, but as a neural stem cell, they give rise to cortical excitatory pyramidal neurons to build a layered cytoarchitecture. From E11 through E13, one RGC will divide into another RGC, as well as an immature neuron that migrate to the deep layers of the cerebral cortex (layers 5 and 6). From E13 through E16, one RGC will divide into another RGC, as well as an intermediate progenitor. These intermediate progenitors will divide and differentiate into neurons that migrate to the upper layers of the cerebral cortex (layers 1–4). After neurogenesis is completed by E16, starting at E17, the RGCs become astrocytes, as well as give rise to oligodendrocyte progenitor cells (OPCs) (Figure 2b).

Mice overexpressing the TS G406R mutation in neural progenitor cells (using *Foxg1^Cre^*) do not show differences in the total number of neurons in the somatosensory cortex at postnatal day 0, suggesting that neurogenesis is not affected by aberrant calcium signaling [122]. In the same study, TS patient-derived neural progenitor cells did not show differences in proliferation *in vitro* [122]. Furthermore, when TS G406R mutant Ca_v_1.2 channels (Ca_v_1.2-G406R) or calcium-impermeable Ca_v_1.2 channels (Cav1.2-G406R-4EQ) were overexpressed into the neural progenitors of the VZ via *in utero* electroporation, both manipulations did not alter the number of RGCs or intermediate progenitors. TS patient-derived *in vitro* cerebral organoids that resemble the GE, from which inhibitory interneurons originate, show no differences in expression of progenitor markers. Together, these studies suggest that Ca_v_1.2 gain-of-function does not lead to changes in neurogenesis of both excitatory and inhibitory neurons of the cerebral cortex. Similar to neurogenesis, mice carrying a heterozygous mutation for Ca_v_1.2-G406R do not show differences in the number of oligodendrocytes as well as the proliferation of oligodendrocyte progenitors [123]. However, whether gliogenesis of astrocytes or microglia are affected by the Ca_v_1.2-G406R gain-of-function mutation remains to be studied.

While the impact of loss of *Cacna1c* (Ca_v_1.2) on neurogenesis is unknown, given that the expression of *Cacna1c* is weak in the VZ throughout mouse embryonic development (see Panagiotakos et al., 2019 [116] for E11, 14, 16, and postnatal day (P)1 and Horigane et al., 2020 [124] for E17) in which the neural progenitors reside, it is likely that loss of *Cacna1c* (Ca_v_1.2) would not impact neurogenesis. Interestingly, male mice and rats with heterozygous loss of *Cacna1c* or male mice with *Cacna1c* loss in excitatory cells show reduced adult neurogenesis in the hippocampus [31,100,125,126]. In contrast, in female rats with heterozygous loss of *Cacna1c* had no effect on adult hippocampal neurogenesis [127]. However, the impact of heterozygous loss of *Cacna1c* on developmental neurogenesis and gliogenesis in the cerebral cortex remains unknown. *Cacna1d* also shows low expression in the VZ at E17 [124], but as there is limited evidence in the cell-type specific expression pattern of *Cacna1d* across developmental stages in the embryonic cortex (see Schlick, Flucher and Obermair, 2010 [128] for whole cortex mRNA expression), the impact of heterozygous loss of *Cacna1d* on cerebral cortical neurogenesis and gliogenesis is difficult to speculate. Nonetheless, similar to mice with heterozygous loss of *Cacna1c*, mice with heterozygous loss of *Cacna1d* also have reduced adult neurogenesis in the hippocampus [129,130].

### Neuronal migration

As neurons are born from neural progenitors, they migrate to their final position in the embryonic cortex and take on a distinct identity. Cortical excitatory and inhibitory neurons use different modes of migration to reach their final location in the developing cortex. Excitatory pyramidal neurons migrate radially from the VZ to either the upper layer or deep layer of the developing cortex (referred to as radial migration; Figure 2A). Inhibitory interneurons that originate from the GE tangentially migrate to reach the embryonic cortex to find their laminar position (referred to as tangential migration [118];; Figure 2A). Both routes of migration require regulation of cell adhesion molecules and actin cytoskeleton. While many mechanisms control transcription factors and cytoskeletal activity during differentiation and migration, calcium signaling is a key component. In particular, calcium activity, measured by a calcium indicator, is increased in migrating neurons when treated with LTCC activators, such as FPL64176 [131,132] or decreased when treated with LTCC blocker nimodipine [131], suggesting LTCC-dependent calcium signaling may play a role in neuronal migration.

Overexpression of wild-type Ca_v_1.2 (Ca_v_1.2-WT) and reporter construct (EGFP) in upper layer excitatory neurons being born (using *in utero* electroporation at E15) leads to more EGFP-expressing neurons in deep layers compared to expression of an empty vector [131]. Furthermore, overexpression of Ca_v_1.2-G406R channels lead to more EGFP-expressing neurons in deep layers compared to the control vector, but also significantly more than when Ca_v_1.2-WT was overexpressed [131]. Similarly, when a Ca_v_1.2 with a different mutation found in TS-like patients (Ca_v_1.2-I1166T) was overexpressed (with a GFP-reporter), in upper layer excitatory neurons being born, more GFP-expressing neurons were found in the deep layers compared to control [133].

Radial migration deficits due to aberrant calcium signaling (via Ca_v_1.2-G406R or Ca_v_1.2-I1166T) were reversed by decreasing calcium permeability of the mutated Ca_v_1.2 channel (Ca_v_1.2-G406R-4EQ [131]; Ca_v_1.2-I1166T-L745P [133]). Furthermore, a TS-like Ca_v_1.2-I1166T mutation-induced migration deficit was reversed when Ca_v_1.2-α_1_ and Ca_v_1.2-β subunits (via W440A mutation) or calmodulin binding (via I1624A mutation) were blocked, but not when calcineurin binding (via A1929P mutation) was inhibited [133]. Interestingly, the Ca_v_1.2-G406R-induced radial migration deficit was observed when the channels were overexpressed prenatally and not when they were expressed postnatally (P1-16) [131], suggesting that radial migration deficits are due to excess embryonic calcium influx in migrating neurons. Also, radial migration deficits were not seen when Ca_v_1.2-G406R was expressed prenatally and blocked early postnatally (via Tet-ON TRE/rtTA system [131], Box 1), suggesting that once calcium influx is normalized, neurons in the wrong laminal location can continue to migrate to their proper location.

While the role of Ca_v_1.2 LTCCs in excitatory cortical neurons are well studied, there are limited studies that indicate the role of Ca_v_1.2 in cortical interneuron migration. *In vitro* studies using forebrain cerebral assembloids (Box 1) derived from human induced pluripotent stem cells (iPSC, Box 1) from TS patients show shorter saltation (each movement of migration) length and increased saltation frequency of inhibitory interneurons [134,135], suggesting inefficient migration of interneurons. Abnormal saltation length (but not frequency) of TS patient interneurons was rescued when treated with an LTCC blocker, nimodipine, suggesting that change in saltation length is due to excess calcium influx [134,135]. TS patient interneurons showed increased expression of GABA receptor subunits (α1, α4, and γ1) and treatment with the GABA antagonist picrotoxin decreased saltation frequency, implicating that saltation length and frequency are differentially regulated. In mice harboring the Ca_v_1.2-G406R mutation (TS2-neo), more inhibitory interneurons migrating to the cortex were observed at E13.5, but the excess migration was not specific to a particular layer [124]. The contrasting results between the two *in vitro* studies [134,135] and *in vivo* study [124] may be due to many factors. One possibility is the difference in developmental expression of exon 8 containing isoforms between the TS2-neo mice [136] and patient-derived neural progenitors. During normal cortical development exon 8A is the predominant isoform until E14, and exon 8 increases in expression significantly by birth [116]. In TS patient-derived neural progenitor cells, exon 8A is significantly higher than exon 8 compared to control patient-derived neural progenitor cells [116]. The TS2-neo mice has an inversed neomycin cassette in exon 8A which leads to reduced expression of both exon 8 and 8A [124,136], whereas the patient-derived assembloids bear the Ca_v_1.2-G406R mutation in exon 8A, which leads to aberrant increase in expression of exon 8A. It is also possible that given the *in vitro* studies and the *in vivo* study measured different parameters (migration speed versus number of neurons) at different timescales (live imaging versus one time point), differences in isoform expression may not be the only contributing factor to the inconsistent results.

The aforementioned *in vivo* studies were conducted in the mouse somatosensory cortex, but not much is known about the developmental role of *Cacna1c* or *Cacna1d* in other cortical regions. One study showed that *Cacna1c* is expressed in the embryonic mouse medial prefrontal cortex (mPFC), and systemic treatment of LTCC blocker nifedipine from E13-16 reduced the number of interneurons by half (though not statistically significant compared to vehicle treated) in the embryonic mPFC [137]. However, as most of the studies on *Cacna1c* and neuronal migration are done in the context of C*acna1c* gain-of-function mutations, it is unclear how loss of *Cacna1c* or *Cacna1d* impacts neuronal migration within the somatosensory cortex as well as other brain regions.

### Neuronal differentiation

As neurons migrate through the developing cortex, they acquire a number of properties that collectively comprise their fate. These properties include their final laminar location, connectivity with other brain regions, and electrical activity. The acquisition of neuronal subtype and laminar location is partly regulated by genetic programs that are specialized for each cell type [138–141]. For example, two types of neurons in layer V are both born simultaneously, but their axon projection targets are different. Neurons that project to the contralateral hemisphere via the corpus callosum (callosal projection neurons, CPNs) require the expression of SATB2, whereas neurons that project to subcortical structures and spinal cord (subcerebral projection neurons, SCPNs) require the expression of FEZF2 and CTIP2 [142–148].

Cortical cultures differentiated from TS patient-derived iPSCs showed significantly more neurons that express upper layer markers and significantly less neurons that express deep layer markers [122]. In particular, there were significantly less SATB2-expressing neurons and more CTIP2-expressing neurons in the upper layer neurons [122]. These *in vitro* results were recapitulated in mice overexpressing the Ca_v_1.2-G406R mutation in neural progenitor cells (using *Foxg1^Cre^*), such that fewer SATB2+ cells were found in deep layers (V and VI) [122]. In agreement with Pasca et al. (2011) [122], another study showed that overexpressing Ca_v_1.2-G406R channels in neurons that are being born at E13 (deep layer neurons) increased the number of CTIP2-expressing neurons and decreased the number of SATB2-expressing neurons [116]. There were no changes in the proportion of CTIP2- and SATB2-expressing neurons when a calcium impermeable Ca_v_1.2-G406R-4EQ channel was overexpressed, suggesting that the higher influx of calcium through mutant Ca_v_1.2 channels contributes to the change in relative abundance of early-born neuron subtypes. Conversely, knocking out *Cacna1c* (Cre-expressing plasmid electroporated into *Cacna1c* floxed mice) at E12 decreased the number of CTIP2-expressing neurons and increased the number of SATB2-expressing neurons [116]. Together, this suggests that Ca_v_1.2 LTCCs play a critical role in the differentiation of neural progenitors to CTIP2- and SATB2-expressing cortical neurons. While the role of Ca_v_1.2 LTCCs in neuronal differentiation was primarily studied in the developing cerebral cortex, results from the Pasca et al. (2011) *in vitro* studies suggest other neuronal subtypes may be altered in TS patients [122]. In TS patient-derived neurons, there was a significant increase in the number of tyrosine hydroxylase (TH)-expressing neurons [122]. This increase in catecholamine synthesizing enzyme was associated with increased catecholamine synthesis in TS patient-derived neurons [122]. However, whether TH expression or catecholaminergic function is altered in mice overexpressing the Ca_v_1.2-G406R mutation in the cerebral cortex or mesencephalic dopaminergic system is unknown. Furthermore, the extent of the impact of LTCCs on neuronal subtype specification across different brain regions remains to be studied.

### Synaptogenesis, axonogenesis, and myelination

Differentiated neurons, as part of their identity, will make unique local and long-range connections through forming synapses with incoming neuronal inputs (synaptogenesis), axons branching out to different brain regions (axonogenesis) and supporting communication via myelination of axons. In mice harboring a heterozygous Ca_v_1.2-G406R mutation, layer 2/3 excitatory neurons in the frontal cortex show a significant decrease in total basal dendritic length and in the number of dendritic branches compared to wild-type mice at P14, but not P7 [149]. Also, mutant mice had approximately six times more neurons with dendritic arbors smaller than 600 um than their littermate wild-type mice [149]. This change in dendritic length and branches was due to activity-dependent retraction of dendrites as seen in primary neurons transfected with Ca_v_1.2-G406R channels and in TS patient iPSC-derived neurons [149]. While the synaptic input frequency and strength to layer 2/3 excitatory neurons are unknown in mice with the G406R mutation, spontaneous calcium transients are increased in cortical neurons expressing Ca_v_1.2-G406R channels during development [131]. It is possible that the shorter dendritic length and branching observed at P14 [149] is an accumulation of increased calcium transients that induced activity-dependent dendritic retraction. Interestingly, treating wild-type mice with LTCC blocker nimodipine at E12.5 or knocking out *Cacna1c* at E12.5 via *in utero* electroporation reduced neurite length [131], suggesting that activity-dependent dendritic retraction seen as a result of TS-Ca_v_1.2 may recruit alternate mechanisms.

Layer 5 cortical neurons in the adult prefrontal cortex of forebrain-specific *Cacna1c* conditional knockout mice (using *CaMKIIa-Cre* [150]) show increased presynaptic excitatory and inhibitory inputs. Also, the relative protein level of VGLUT1 was higher than VGAT (more excitatory inputs than inhibitory inputs) [150], which may suggest the net effect on pyramidal neurons is increased excitatory inputs. It is possible that increased excitatory inputs may lead to activity-dependent retraction of dendrites. Interestingly, treating with LTCC agonist FPL64176 marginally rescued the neurite reduction seen in *Cacna1c* KO condition, which is speculated to be via Ca_v_1.3 stimulation [131]. In fact, heterozygous loss of *Cacna1d* in new-born neurons of the hippocampus show shorter dendritic branch length [130], suggesting that *Cacna1d* may be critical for dendritic branching during development in other brain regions. Together, these studies suggest that aberrant LTCC expression or channel function in developing cortical neurons impacts their dendritic morphology, which in turn will affect their communication with other brain regions.

Similar to dendritic morphology changes, axon projections are also altered with Ca_v_1.2 LTCC mutations. In callosal projection neurons (neurons that make axonal projections from one side of the hemisphere to the other) with excessive WT Ca_v_1.2 channels at E15.5 (when upper layer neurons are born), there is a reduction in axon arborization (i.e. spread of the axons) in layers 1–3 and layer 5 [133]. Furthermore, Ca_v_1.2 channels with the TS-like I1166T mutation reduced axon arborization in layers 1–3 and layer 5, but only layers 1–3 showed a stronger reduction compared to when overexpressing Ca_v_1.2-WT channels [133]. The reduced arborization was not due to a change in axon density in white matter, i.e., number of projections received from the contralateral hemisphere, suggesting that axon guidance during development is not perturbed in callosal projection neurons with excessive calcium influx. The aberrant axon arborization as a result of Ca_v_1.2 WT and TS-like Ca_v_1.2 I1166T mutation overexpression was partially rescued only in layers 1–3 when calcium permeability was reduced (L745P mutation) and Ca_v_1.2-α_1_ and Ca_v_1.2-β subunits (via W440A mutation) were blocked [137]. However, it remains unknown whether the TS Ca_v_1.2-G406R mutation or loss of *Cacna1c* or *Cacna1d* impacts axonogenesis.

Similar to neurons, in TS2-neo mice that express G406R mutant channels, oligodendrocyte progenitor cells (OPCs) show increased calcium influx at baseline and show higher extension of processes [123]. Interestingly, unlike neurons that show decreased dendritic morphology, there were more OPCs that showed more complex morphology (more branching) in TS2-neo compared to control mice. This increased proportion of mature OPCs may underlie the increased myelination observed in TS2-neo mice in the corpus callosum [123]. Additionally, the increase in myelination is not due to an increased number of OPCs or oligodendrocytes, as their numbers in TS2-neo mice were comparable to control mice. Whether the increase in myelination underlies social and cognitive behavioral deficits see in TS2 mice is unclear, increased myelination has been observed in ASD [151–153] and seen in mice with ASD-like behavioral deficits [154,155]. In addition, the effect of loss of *Cacna1c* on myelination remains to be examined.

The role of LTCCs in synaptogenesis, axonogenesis, and myelination has only been studied in gain-of-function or loss-of-function in *Cacna1c* and not in *Cacna1d*. Three *de novo* mutations in *CACNA1D* have been associated with autism (p.A749G and p.G407R, see Pinggera et al., 2015 [24]; S652L, see Hofer et al., 2020 [156]), and these mutations in HEK293 cells (human embryonic kidney cells) show changes in their electrophysiological properties comparable to gain-of-function changes. Therefore, it is possible that introducing these *Cacna1d* mutations *in vivo* or *in vitro* in neurons may show similar changes in synaptogenesis and axonogenesis as *Cacna1c* gain-of-function studies have reported.

### Ca_v_1.2 and Ca_v_1.3 outside of the cerebral cortex

*Cacna1c* and *Cacna1d* expression across development is not restricted to the cerebral cortex, but also the hippocampus and cerebellum, and all three brain regions show a similar developmental expression pattern [128]. This suggests that mutations in *Cacna1c* or *Cacna1d* in the hippocampus or cerebellum may show similar deficits as the manipulations done in the cerebral cortex. In fact, loss of *Cacna1c* in cerebral cortical and hippocampal excitatory neurons (using *Emx1^IRES-^^Cre^*) leads to smaller cerebral cortex and hippocampal volume [157], but it is unclear whether the change in volume is due to differences in cell number or dendritic arborization. As the thickness of the somatosensory cortex was not different [157], it is likely that loss of *Cacna1c* affects dendritic arborization that may result in volumetric changes. Nevertheless, the exact role of *Cacna1c* and *Cacna1d* in the development of the hippocampus and cerebellum as well as other brain regions that may express these channels during development remains to be studied.

### Timing of Ca_v_1.2 or Ca_v_1.3 mutations and behavioral consequences

Behavioral changes associated with Ca_v_1.2 and Ca_v_1.3 mutations may be dependent on the timing at which the mutation occurs during brain development and maturation. Embryonic deletion of *Cacna1c* in postmitotic forebrain glutamatergic neurons (*Nex-Cre*) promotes neuropsychiatric disorder-associated phenotypes, such as cognitive decline, impaired synaptic plasticity, reduced sociability, hyperactivity, and increased anxiety [2]. Additionally, this embryonic deletion increases the susceptibility to chronic stress, suggesting that Ca_v_1.2 interacts with the environment to shape later disease vulnerability [2]. This is recapitulated in humans through the interaction of *CACNA1C* SNPs with adverse life events to later alter the risk of developing symptoms of a neuropsychiatric disorder, seen specifically in the interaction of *CACNA1C* and adult trauma to predict depression symptoms in humans [2]. However, this phenotype is not seen when *Cacna1c* is deleted in glutamatergic neurons in adulthood (tamoxifen administration in postnatal weeks 11–13 in *Camk2a-CreER* line) [2], suggesting that *Cacna1c* mutation-related behavioral changes are developmentally driven. Similarly, mice with heterozygous loss of *Cacna1c* show anxiety-like phenotype, impaired social behavior, learning, and memory [150] but mice with *Cacna1c* deleted in adult forebrain glutamatergic neurons only exhibit anxiety-like phenotype and social deficits [150,158].

## Conclusions

From the studies described in the genomic regulation section of this review, it is clear that neuropsychiatric disease-associated SNPs within non-coding regions of *CACNA1C* have the potential to impact gene expression by disrupting a variety of mechanisms that are normally recruited during transcription. These include (1) structural changes in chromatin (such as looping) and (2) epigenetic changes that include DNA methylation, histone modifications, regulation by microRNAs, and splicing. Thus, even though the significance of intronic SNPs to disease has been a question, the recent focus on understanding basic mechanisms of transcriptional regulation at the genomic level will allow us to probe how intronic SNPs (like those found in neuropsychiatric and neurodevelopmental disease-linked genes *CACNA1C* and *CACNA1D*) can impact gene expression. From the studies described in the brain development section, it is evident that alterations in either levels of Ca_v_1.2 and Ca_v_1.3 channels through altered gene expression or in function of these channels can impact certain aspects of neuronal development. Given the compelling evidence for a role for calcium signaling in brain development, the time is prime to further explore the role of Ca_v_1.2 and Ca_v_1.3 channels in brain development to connect the dots between genetic aberrations, impact on the brain, and disease-associated behaviors.
